# Pattern Classification of Large-Scale Functional Brain Networks: Identification of Informative Neuroimaging Markers for Epilepsy

**DOI:** 10.1371/journal.pone.0036733

**Published:** 2012-05-17

**Authors:** Jie Zhang, Wei Cheng, ZhengGe Wang, ZhiQiang Zhang, WenLian Lu, GuangMing Lu, Jianfeng Feng

**Affiliations:** 1 Centre for Computational Systems Biology, Fudan University, Shanghai, People’s Republic of China; 2 Fudan University-JinLing Hospital Computational Translational Medicine Centre, Fudan University, Shanghai, People’s Republic of China; 3 Mathematical Department, Zhejiang Normal University, Jinhua, Zhejiang Province, People’s Republic of China; 4 Department of Radiology, JinLing Hospital of Nanjing, People’s Republic of China; 5 Department of Computer Science, Warwick University, Coventry, United Kingdom; Wake Forest School of Medicine, United States of America

**Keywords:** **Keywords**: functional brain networks, pattern classification of brain networks, brain asymmetry, epilepsy

## Abstract

The accurate prediction of general neuropsychiatric disorders, on an individual basis, using resting-state functional magnetic resonance imaging (fMRI) is a challenging task of great clinical significance. Despite the progress to chart the differences between the healthy controls and patients at the group level, the pattern classification of functional brain networks across individuals is still less developed. In this paper we identify two novel neuroimaging measures that prove to be strongly predictive neuroimaging markers in pattern classification between healthy controls and general epileptic patients. These measures characterize two important aspects of the functional brain network in a quantitative manner: (i) coordinated operation among spatially distributed brain regions, and (ii) the asymmetry of bilaterally homologous brain regions, in terms of their global patterns of functional connectivity. This second measure offers a unique understanding of brain asymmetry at the network level, and, to the best of our knowledge, has not been previously used in pattern classification of functional brain networks. Using modern pattern-recognition approaches like sparse regression and support vector machine, we have achieved a cross-validated classification accuracy of 83.9% (specificity: 82.5%; sensitivity: 85%) across individuals from a large dataset consisting of 180 healthy controls and epileptic patients. We identified significantly changed functional pathways and subnetworks in epileptic patients that underlie the pathophysiological mechanism of the impaired cognitive functions. Specifically, we find that the asymmetry of brain operation for epileptic patients is markedly enhanced in temporal lobe and limbic system, in comparison with healthy individuals. The present study indicates that with specifically designed informative neuroimaging markers, resting-state fMRI can serve as a most promising tool for clinical diagnosis, and also shed light onto the physiology behind complex neuropsychiatric disorders. The systematic approaches we present here are expected to have wider applications in general neuropsychiatric disorders.

## Introduction

Neuropsychiatric disorders, whose rank in frequency is second only to cardiovascular disease, are widespread all over the world. A large percentage of the population will experience some type of neuropsychiatric disorders at some stage in their life. Traditionally, neuropsychiatric diagnosis is based on a categorical taxonomy arrived at from clinical observations, and questionnaires developed with the aid of rating scales. The results have sometimes been reported to be inconsistent as the questionnaire filled by the subject tends to be subjective. Over the past decade, clinical doctors and researchers have become increasingly interested in finding highly predictive neuroimaging markers that can provide objective ways to predict and evaluate neuropsychiatric conditions [1], [2]. With the recent advances in functional magnetic resonance imaging (fMRI), which can provide an unprecedented opportunity to map large scale brain connectivity [3], [4], [5], [6], it remains an important problem to know whether resting state fMRI contains sufficient information to aid the diagnosis of general neuropsychiatric disorders. In practice, the advantage of fMRI is its high spatial resolution, which is beneficial to source location in epilepsy. In comparison, electroencephalogram, which is widely used in clinical diagnosis of epilepsy, has a very high temporal resolution but a limited spatial resolution.

The human brain can be deemed as a large-scale network, with nodes being distinct brain regions and edges representing functional connectivity among them. It has been suggested that many functional brain disorders, such as depression, Alzheimer’s disease, schizophrenia and autism can be described as dysconnectivity syndromes, which are related to the disruption of the connectivity patterns among spatially distributed regions of the brain that underlie normal functioning [7], [8], [9], [10]. Recently, a large number of multivariate methods and pattern recognition approaches [11], [12] have been applied to complicated spatial-temporal patterns of fMRI data, with the ultimate goal of diagnostic classification of various brain disorders, ranging from depression [7], Alzheimer’s disease [13], attention deficit hyperactivity disorder [14] to schizophrenia [15].

Most current research on fMRI simply focuses on describing group differences between subject classes (knowing the label of each subject) using a relatively small number of subjects. This cannot classify or predict the brain behavior across individuals. In this paper, we address the problem of accurately classifying the brain state (healthy or with neuropsychiatric disorders) on an individual basis for a large data set. This is generally a complicated endeavor that must be approached with sensitive neuroimaging markers and efficient feature-selection methods. The neuropsychiatric disorder we focus on here is epilepsy, which is caused by abnormal neural discharge in the cortex. Epilepsy is one of the most common neuropsychiatric disorders, affecting about 50 million people in the world [16], [17], [18]. Traditionally, the amplitude of low frequency fluctuations [19] and regional homogeneity [20] were used to study the change in blood-oxygen level dependent (BOLD) signals. Although these approaches can spot the regional change in the brain, they ignore the dynamic interactions among the distributed brain areas. At the network level, the default mode network (DMN) [21], [22] and other networks [23], [24] have been found to demonstrate abnormalities for different kinds of epileptic patients. Currently, much work was confined to empirically chosen brain regions or subnetworks, and a global exploration of the whole functional brain network as well as its application in individual pattern classification are expect to provide more information and diagnostic tools [25], [26], [27]. In this paper, we proposed two efficient neuroimaging markers at both local and global level of the functional brain network, which are proved to be highly sensitive biomarkers in general epilepsy prediction and can shed lights onto the neuro-pathophysiological mechanism of epilepsy. In particular, we develop a distinct, global brain asymmetry measure that has not been previously exploited in brain disorder classification. With the proposed neuroimaging markers, our goal is to develop a systematic and accurate pattern classification methodology for large-scale functional brain network discrimination for epilepsy and possibly other neuropsychiatric disorders.

## Materials and Methods

### Participants

There are altogether 80 healthy controls (age: 24.89
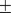
8.63) and 100 epileptic patients (age: 23.85
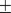
5.66). All subjects are right handed. The criteria for selection of epileptic patients are that the patients had unprovoked seizure for more than two times, and had typical symptoms. The patients enrolled in our study have different kinds of epilepsy (e.g., temporal lobe epilepsy, partial and global epilepsy). Statistical tests show that differences in age between these two groups is not significant (p<0.05). In the patient group, there are: a) 18 global seizure patients and 70 partial seizure patients. b) 82 patients use antiepileptic drugs, and 18 without medications. The patients who were treated with anti-epileptic drugs (AEDs) use valproic acid, phenytoin, carbamazepine and topiramate. Written informed consent was obtained from all participants. The study was approved by the local medical ethics committee at Jinling Hospital, Nanjing University School of Medicine.

### Data Acquisition and Preprocessing

All data were collected on a 3 Tesla Siemens Trio Tim scanner with an eight channel phased array head coil. Resting state fMRI data were acquired axially by using an echo-planar imaging (EPI) sequence. The following parameters were used: TR/TE = 2000 ms/30 ms, FA = 90°, matrix = 64×64, FOV = 24×24 cm^2^, slice thickness = 4 mm, and slice gap = 0.4mm. A total of 30 slices were used to cover the whole brain. Each section contained 250 volumes. Subjects were instructed to relax, hold still, keep their eyes closed without falling asleep, and think of nothing in particular. Routine anatomical MRI data were acquired to detect structural abnormality. T1-weighted image parameters: TR/TE = 350 ms/2.46 ms, FA = 90°, matrix = 320×256, FOV = 24×24 cm^2^, and slice thickness = 4 mm, slice gap = 0.4 mm, and a total of 30 slices were acquired. T2-weighted image parameters: TR/TE = 4000 ms/98 ms, FA = 120°, matrix = 512×307, FOV = 22×20 cm^2^, and slice thickness = 4 mm, slice gap = 0.4 mm, a total of 30 slices were acquired. Coronal T2-FLAIR-weighted image parameters: TR/TE = 7000 ms/87 ms, FA = 150°, matrix = 256×256, FOV = 24×19.5 cm^2^, and slice thickness = 4 mm, slice gap = 0 mm, a total of 28 slices were acquired.

We perform data-preprocessing using the software DPARSF. DPARSF is based on some functions in Statistical Parametric Mapping (SPM) and Resting-State fMRI Data Analysis Toolkit (REST), and it has integrated basic preprocessing steps in a convenient way. First slice-timing adjustment and realignment for head-motion correction were performed, then we use standard Montreal Neurological Institute (MNI) template provided by SPM2 for spatial normalization (resampling voxel size: 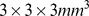). After smoothing (*FWHM* = 8 *mm*), the BOLD signals were filtered (band pass, 0.01∼0.08 Hz) to remove very low-frequency drift and high-frequency noises (like cardiac and respiratory rhythms). The following variables are regressed out as covariate for each voxel in the data pre-processing: 1. 6 head motion parameters. 2. Global mean signal. 3. White signal. 4. Cerebrospinal fluid signal. The registered fMRI time series were segmented into 116 regions (90 from cortex and 26 from cerebellum) using the anatomically labeled template by Tzourio-Mazoyer et al. [28] For each brain region, its representative fMRI time series, or BOLD signal, is obtained by averaging the fMRI time series of all voxels in that region. In practice, a component base noise reduction method [29] can also be applied if noise is significant. Finally, for each subject, there is a set of 116 BOLD signals where *x_i_*(*t*) *i = *1, 2, …, 116 represents the BOLD signal in the *i*th brain region.

The head movements of the subjects can have some effect to the functional connectivity (e.g., lead to spurious connectivity [30], [31]). Here all the subjects enrolled in our study have very small head movements (translations<1 mm for all subjects; rotations<1° for all subject except two patients, and these two patients’ rotations are smaller than 1.5°), which have been regressed out in data preprocessing. We have furthermore performed two sample t-test to the 6 head movement parameters (a rigid body transformation in 3 dimensions is defined by 6 parameters: 3 translations and 3 rotations) for the healthy subjects and the epileptic patients, and find that there is no significant difference between the two groups.

### Neuroimaging Marker I: Functional Integration by Community Matrix K

The functional connectivity of the brain network is usually measured by cross-correlation between regional BOLD time series. The functional brain network so obtained, however, can be quite dense, which usually degrades the performance of pattern classification approaches. More importantly, the cross-correlation matrix does not characterize the community structure of the network explicitly, i.e., whether two brain regions belong to the same functional cluster or not, a feature that captures coordinated behavior among distributed brain regions known as functional integration [32].

**Figure 1:**
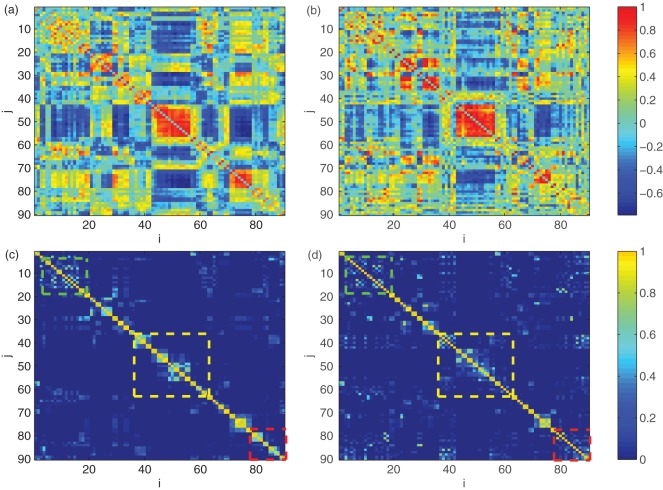
Visualization of Cross-Correlation matrix (a and b) versus Community matrix *K* (c and d) for the same healthy control (left column) and epileptic patient (right column). From **c** and **d** we can see that the pixels distributed near the main diagonal are much brighter in healthy subjects than those in patients, as are highlighted by the three boxes, which cannot be observed in cross-correlation matrix (**a** and **b**). These pixels mostly correspond to the functional connections across the two hemispheres.

To overcome this shortcoming, we propose a novel, adaptive metric called the community matrix *K* based on a method known as *k*-means clustering [33], which can reflect the community structure of the brain network in a sparse manner, see Figure 1. We consider a matrix *K* whose (*i,j*) th entry is an estimate of the probability that the *i*th and the *j*th brain region belong to the same functional community. The basic steps of calculating the community matrix *K* are:

1. Initialize k centroids by randomly choosing k data points;
2. Assign each data point to the closest centroid according to the Euclidean distance Uij = |xi − xj|2;
3. For each cluster compute its mean as the new centroid;
4. Repeat Steps 2 and 3 until the centroids no longer move.

For each run of *k*-means clustering, we get a matrix *K* with *K*(*i, j*) being 1 only if *x*_i_ and *x_j_* are assigned to the same community and 0 otherwise. By averaging *K* over *L* trials (we choose *L* = 500 so that the result is stable), we obtain a community matrix *K*, with *K (i,j)* being the probability that the *i*th and the *j*th brain region belong to the same functional community, reflecting the functional integration of the overall cortex. Here we set the number of clusters to be relatively large (*k* = 30) so that *K* is sparse. This way only those regions that are highly cooperative will be assigned to the same cluster. Empirically, *K* demonstrates consistent connectivity patterns for *k* within a large range of values (from 15 to 45), see Text S1, Figure S1 and Figure S2 for details.

### Feature Selection of *K* via Sparse Regression

The community matrix *K* has a large dimension: for 90 brain regions, there would be 90*(*90*−1)/2* = *4005 edges, which can lead to the “curse of dimensionality” problem in classifier. Usually, only a small proportion of the pathways in the brain might be responsible for the dysfunction of the brain network, so, the effective dimensions in *K* might be small. Here we use the state-of-the art feature-selection technique called sparse regression [33] to extract the key features (i.e., the most discriminative edges) from *K*.

Sparse modeling is a rapidly developing area at the intersection of statistics, machine-learning and signal processing. It can expose highly predictive patterns or signatures, (i.e., a small number of the most relevant variables in a high-dimensional feature space) and is most appealing for practical disease marker identification [34], [35]. In our case, sparse regression can be used to identify a small proportion of the edges in the matrix *K* (which are the key features in pattern classification), shedding important light onto the affected functional pathways and brain regions of epileptic patients.

The details of sparse regression technique can be found in Text S2. Basically, by formulating the feature matrix of the training set (each row is a 4005 dimensional feature-vector *a* from one subject) and the label of subjects (1 for healthy and −1 for patients) into a linear regression, sparse regression returns a regression coefficient vector *x* (4005 dimensional), each entry of which (the absolute value) indicates the contribution of the corresponding feature to discriminating the two groups. It can provide effective feature selection even when the number of training subjects (90) is much lower than the number of features (4005). Furthermore, we apply a random sampling in sparse regression, which can preserve a group of relevant features that, combined, will possess even higher discrimination power. This way, the correlation among different features are taking into full account and utilized, which is superior to considering each feature separately (such as independent multiple t-test).

### Neuroimaging Marker II: Global Connectivity Asymmetry of Equivalent Brain Regions

It is well known that the cerebral cortex exhibits marked structural symmetry across the left and right hemispheres, but is clearly asymmetrical with regard to function or physiology. The left hemisphere is normally dominant in language and logical processing, whereas the right hemisphere is dedicated to spatial recognition [36], [37]. Most work on brain asymmetry focuses on anatomical structures (such as the morphometric change of cortex) using modern imaging techniques. The investigation of functional asymmetry, on the other hand, is traditionally based on cognitive studies (e.g., handedness and language ability) of patients with unilateral lesions or split-brain surgery [38]. Generally, there are few studies of asymmetry measures, based on functional interactions among brain regions [39], [40], [41]. Furthermore, the exact characterization of asymmetry of equivalent brain regions in terms of global, functional connectivity patterns, and its application to brain disorder classification has not been reported. Here we propose a new, quantitative, asymmetry index termed ***g***lobal connectivity asymmetry (GCA) of bilaterally homologous brain regions, which is expected to provide a more fundamental characterization of overall left-right asymmetry at the network level. Since the cortex in each hemisphere is divided into 45 regions, there are 45 pairs of bilaterally homologous brain regions in the cortex.

**Figure 2:**
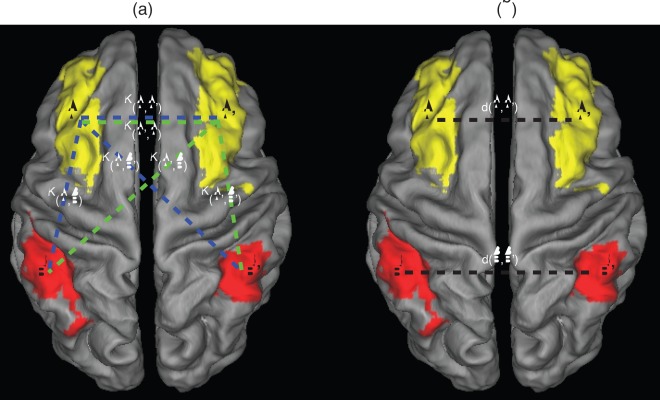
Illustration of two asymmetry measures using a pair of equavelent regions A and A’ for demonstration. a global connectvity asymmetry measure *ρ.* The connectivity profile of A (i.e., vetor [*K*(A,A’), *K*(A,B’), *K*(A,B)]) and A’ (vector [i.e., *K*(A’,A), *K*(A’,B), *K*(A’,B’]) are represented by dashed lines of bule and green, respectively. Note that the connectivity *K*(A,A’) equals *K*(A’,A), and are both ploted for clarity. **b** pairwise brain-region synchronization *d*, which is the standardized Euclidean distance between BOLD signals from equavalent brain regions A and A’, B and B’, respectively (by black dashed lines).

The global connectivity asymmetry is defined by the degree of dis-similarity between the connectivity profiles of two bilaterally homologous brain regions. The connectivity profile of region *i* indicates the global pattern of connectivity of region *i* to the rest of cortex, and is defined as the *i*th row in community matrix *K* (i.e., *K*(*i*,:), see Figure 2a). We find that a useful quantitative measure of the asymmetry between bilaterally homologous brain regions *i* and *j* is 1 minus the correlation coefficient between *K*(*i*,:) and *K*(*j*,:), hence we define an asymmetry index as:
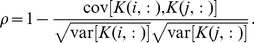
If two bilaterally homologous regions (*i* and *j*) are functionally connected to the whole cortex in a similar way, then there will be a large correlation coefficient between *K*(*i*,:) and *K*(*j*,:), leading to a small *ρ*, indicating a low level of asymmetry. On the contrary, if region *i* and *j* interact with other regions in a very different manner, this will result in a large *ρ*, i.e., a high level of asymmetry. Basically, *ρ* measures asymmetry of two bilaterally homologous brain regions in terms of their functional interaction and information transmission to other parts of the brain. Note that *ρ* is 45-dimensional since there are 45 pairs of equivalent brain regions in the cortex.

Another asymmetry measure we propose is termed the “*pairwise brain-region synchronization*” *d*, which we define also for bilaterally homologous brain regions as the standardized Euclidean distance between the corresponding regional BOLD time series, see Figure 2b. The reason why we use standardized Euclidean distance (see Text S3 for definition) rather than Euclidean distance here is to eliminate the nonstationary effect of the time series. The human brain consists of the left and right hemispheres that are connected by a bundle of neural fibers called the corpus callosum. In normal brain function, the two hemispheres work together, communicating and sharing information across the corpus callosum. Here *d* reflects the callosal information transfer in the brain, and a large *d* indicates weak synchronization/information transfer between a pair of bilaterally homologous brain regions, and therefore stronger asymmetry.

## Results

### Functional Integration: Community Matrix K

The community matrix *K* reflects the functional integration among distributed brain regions in a sparse manner, thereby serving as a unique “neuro-signature” of the brain state. An example of the cross-correlation matrix and community matrix *K* for a typical healthy control and an epileptic patient is shown in Figure 1. As can be seen, the connectivity in the former is too dense to reveal significant difference in functional connectivity patterns. While for the community matrix which manifests only the significant pathways among highly cooperative regions, the difference is more evident. We find that the healthy subjects demonstrate marked community structure near the main diagonal, indicating strong coherence in neuro-activities across two hemispheres.

### Altered Functional Connectivity Patterns in Epileptic Patients

**Figure 3:**
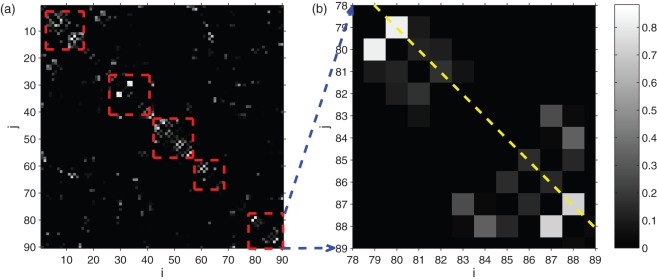
Visualization of group difference in terms of community matrix *K*, b is an enlargement of a. The white pixels correspond to edges in *K* that are more discriminative across the two groups (these edges have a larger regression coefficient in sparse regression). Specifically, the second main diagonal (as is indicated by a yellow dashed line in **b**) contains connectivities between pairs of bilaterally homologous brain regions. In figure **b**, the connectivities between brain region 79 and 80 (left and right heschl), 87 and 88 (left and right temporal-pol-mid) are most significant. Here we highlight most of the discriminative edges in by 5 red boxes, which belong to frontal, limbic, occipital, parietal, and temporal lobe, respectively.

Figure 3 demonstrates the group difference between the healthy subjects and epileptic patient in terms of their community matrix *K*. We rank the all the edges in the network according to the corresponding regression coefficient in sparse regression. A larger regression coefficient (i.e., whiter pixels) corresponds to edges that are more discriminative across the two groups. Although the patient group includes different kinds of epilepsy, a common feature, as shown in Figure 3a, is that the most discriminative edges (i.e., whiter pixels) are near the main diagonal, corresponding to functional pathways across the left and right hemispheres. Specifically, many white pixels lie on the second main diagonal (Figure 3b), corresponding to functional connectivity between bilaterally symmetric brain regions (i.e., between region 1 and region 2, region 3 and region 4,…). To fully understand the change in neuro-circuitry in epileptic patients, we list the 20 edges with the largest regression coefficient (absolute value) in Table 1 and categorize them into two kinds: 1. decreased connectivity in patients (compared to healthy controls), including A) 5 edges from of bilaterally symmetric regions (heschl, fusiform, temporal-pole-mid, amygdale and occipital-mid), which indicates that the inter-hemispheric connection are impaired. B) 4 edges between middle cingulum and insula (both uni- and bilateral). These connections are shown to be involved in environmental monitoring and skeletomotor body orientation [42]. The decrease of these functional connectivities is going to affect the response selection and action of the patients. C) 2 edges from super_marginal to fronal_inf_oper. 2. Increased connectivity in patients (compared to healthy controls), including D) 3 edges within frontal lobe (among inferior, middle and superior part) and 1 edge between inferior and superior parietal lobe; E) 2 edges between cuneus and calcarine (unilateral). F) 2 edges from subcotical area (amygdale, insular and caudate). Our finding on both the decreased and increased functional connectivity indicates that epilepsy is associated with imbalance of excitatory and inhibitory pathways in the brain [24].

**Table 1:** Top 20 links (according to the amplitude of regression coefficient *x*) among the selected 400 edges in community matrix *K*.

Brain Region	Brain Region	Regression coefficient	*P* value
Cingulum-Mid-L	Insula-L	+0.8827	2.947 *1e-5
Frontal-Inf-Tri-L	Frontal-Inf-Oper-R	−0.8447	0.0042
**Heschl-R**	**Heschl-L**	+0.8178	4.0817*1e-8
Cingulum-Mid-R	Insula-R	+0.8014	4.9377*1e-6
Cingulum-Mid-R	Insula-L	+0.8009	7.4975*1e-6
Cuneus-R	Calcarine-R	−0.7683	0.0029
Cingulum-Mid-L	Insula-R	+0.7503	1.8627*1e-5
**Fusiform-R**	**Fusiform-L**	+0.7421	**2.2000e-009**
**Temporal-Pole-Mid-R**	**Temporal-Pole-Mid-L**	+0.7257	1.780*11e-7
Frontal-Mid-R	Frontal-Sup-Orb-R	−0.7012	0.0096
Parietal-Inf-L	Parietal-Sup-R	−0.6785	0.0020
SupraMarginal-R	Frontal-Inf-Oper-R	+0.6691	0.0002
Postcentral-R	Precentral-R	−0.6670	0.0014
**Amygdala-R**	**Amygdala-L**	+0.6665	5.8764*1e-7
**Occipital-Mid-R**	**Occipital-Mid-L**	+0.6434	1.1399*1e-6
Temporal-Inf-R	Caudate-R	−0.6024	0.0067
Amygdala-R	Insula-R	−0.5695	0.0002
Frontal-Inf-Orb-R	Frontal-Inf-Oper-L	−0.5622	0.0061
Cuneus-L	Calcarine-L	−0.5578	0.0015
SupraMarginal-L	Frontal-Inf-Oper-L	+0.5462	0.0027

The brain regions that are involved in each of these 20 edges are listed in the first two columns. Among these links, 7 links are from right-hemisphere, 5 are from symmetric left- and right-hemisphere (bold), 5 are from nonsymmetric left- and right-hemisphere, 3 remaining are from left-hemisphere alone. The 3^rd^ column is the regression coefficient, with the sign of the group difference (Healthy minus Patient). The 4^th^ column is the *P* value of the edge by ranksum-test (we use ranksum-test as the distribution of some of the edges in community matrix *K* is not Gaussian). We find that 9 edges (in bold) are statistically significant (*p* = 0.05, ranksum-test, with Bonferroni correction. Here the single edge threshold is 0.05/650 = 7.69*1e-5, in which 650 is the average number of non-zero edges in *K*. The number of non-zero connections is obtained by counting the number of entries in the average community matrix *K* that are greater than 0.05).

Finally we summarize these altered functional connections in terms of the established 6 resting state networks (RSNs) in human brain, each with specific anatomic pattern and a corresponding function [43]. We find 1 edges belong to default network, 4 belong to dorsal attention network, 4 belong to visual network, 1 belong to auditory network, 1 belong to sensory network, and 2 belong to the subcortical network. The rest 7 edges are inter-network edges. From this we can see that the function related to all 6 resting state networks are affected in epileptic patients (especially in the dorsal attention network and subcortical network). These altered functional connections may underlie the pathophysiological mechanism of the impaired cognition functions of the brain.

### Enhanced Asymmetry of Functional Brain Networks

**Figure 4:**
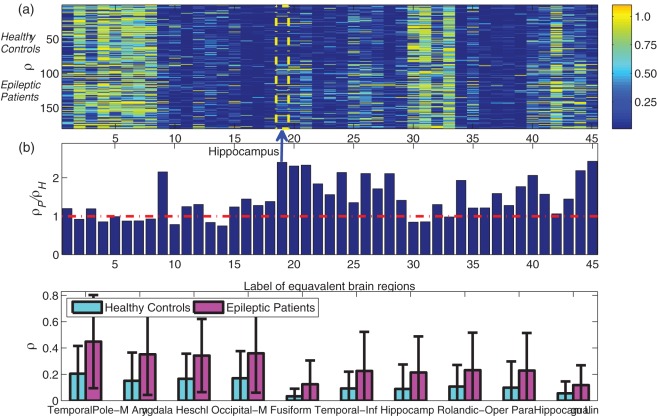
Asymmetry measure *ρ* for 45 pairs of equivalent brain regions a, Visualization of *ρ* for all subjects, with each row corresponding to a subject, and each column represents each pair of equivalent brain regions. The top 80 rows are healthy controls, and the bottom 100 rows are epileptic patients, separated by a black dashed line. A large *ρ* indicates a high level of asymmetry. b, The ratio between group mean value (*ρ_*P/*ρ_*H) for each pair of brain regions. The red dashed line corresponds to *ρ_*P/*ρ_*H = 1, i.e., the two groups have the same group mean value. The most asymmetric brain region according to *ρ_*P/*ρ_*H (i.e., amygdale), is highlighted. c, The 10 most discriminative regions across the two groups according to *P* value of two sample *t*-test, with the mean and standard deviation of *ρ* being shown for the two groups. The corresponding *P* values are (unit: 10^−3^): 0.0002, 0.0017, 0.0027, 0.0031, 0.0234, 0.2620, 0.6166 0.6511, 0.7256, 1.1926. Other significantly changed regions (*P*<0.01/45) include Occipital_Inf, Temporal_Sup, Parietal_Inf, Temporal_Mid, Calcarine, and Frontal_Mid. As is shown, *ρ* is much larger for epileptic patients than for healthy controls.

**Figure 5:**
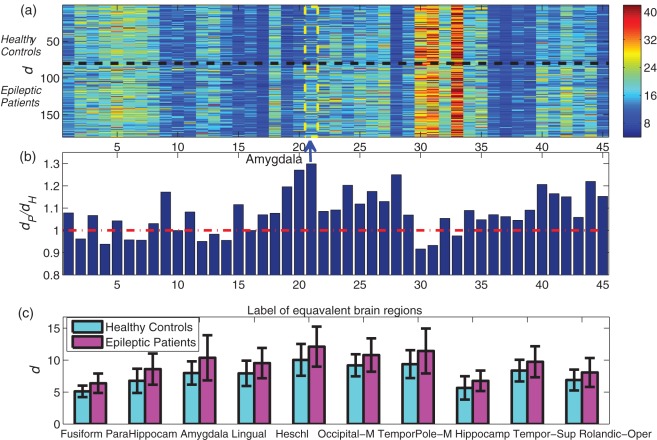
Pairwise brain-region synchronization *d* for 45 pairs of equivalent brain regions a, Visualization of *d* for all subjects, with each row corresponding to every subject, and each column represents each pair of equivalent brain regions. The top 80 rows are healthy controls, and the bottom 100 rows are epileptic patients, separated by a black dashed line. A large *d* indicates weak synchronization between a pair of brain regions and thus a high level of asymmetry. **b**, The ratio between group mean value (*d_*P/*d_*H) for each pair of brain regions. The red line corresponds to *d_*P/*d_*H = 1, i.e., the two groups have the same group mean value. The most asymmetric brain regions according to *d_*P/*d_*H (i.e., hippocampus) is highlighted. **c**, The 10 most discriminative regions across the two groups according to P value of two sample t-test, with the mean and standard deviation of *d* being shown for the two groups. The corresponding *P* values are (unit: 10^−3^): 0.0000004, 0.0001, 0.0001, 0.0028, 0.0032, 0.0044, 0.0086, 0.0252, 0.0267, 0.1217. As is shown, *d* is much larger for epileptic patients than for healthy controls.

Figure 4a shows the global connectivity asymmetry *ρ* for 45 pairs of bilaterally homologous brain regions for all subjects: each row represents a subject, and each column corresponds to a pair of brain regions. For a given pair of such regions, we then determine the average asymmetry of (i) healthy controls (mean value *ρ*_H) (ii) epileptic patients (mean value *ρ_*P), and this is summarized by the ratio *ρ_*P/*ρ_*H in Figure 4b. Since a large *ρ* indicates a higher level of asymmetry between a pair of equivalent brain regions, a ratio significantly larger than 1 (i.e., above the red line in Figure 4b) indicates highly asymmetric brain region connectivities in patients compared with healthy controls. It may be seen that the asymmetry of patients increases significantly in multiple brain regions, with the 10 pairs of most asymmetric regions shown in Figure 4c. Figure 5 presents the results for pairwise brain-region synchronization *d* in a similar manner as Figure 4, and we find that Figure 4b and Figure 5b demonstrate quite similar patterns, suggesting these two asymmetry measures *ρ* and *d* are highly correlated, i.e., a larger *d* (i.e., weak synchronization) between symmetric brain regions corresponds to an increased level of asymmetry.

### Classification Accuracy: Distinguish between Healthy Controls and Epileptic Patients

The next step to neuroimaging markers identification is the classification, for which we use support vector machine (SVM) as the classifier [12]. The details of the SVM classifier can be found in Text S4. First we took 50% of the whole dataset as the training set for feature selection and SVM classifier training (note that we are using 50% as training set, which is theoretically more difficult than using a larger percentage, say 60% or 80% as training set). To test the generalization performance of SVM, the rest of the data set, i.e., those not previously presented to the classifier, are put to the trained SVM using the selected features for cross-validation. An averaged classification accuracy of 77.6% is achieved (average over 100 trials) with the selected, most significant 400 edges in *K* matrix (we use 400 here; it is stable with 300 to 600, with the corresponding results shown in Figure S3). Then we tested how much prediction power the asymmetry measures *ρ* and *d* possess. For *ρ* and *d*, an average accuracy of 75.5% and 75.8% were obtained, respectively (over 100 trials). From these results, we can see that the community matrix *K* and asymmetry measures are proved to be neuroimaging markers with highly predictive power. Remarkably, by merging the 45-dimensional asymmetry feature *ρ* with the 400-dimensional feature selected from *K* matrix (50% training set), we can achieve an accuracy of 80.2%. Most prominently, using leave-one-out prediction (i.e., the classifier was trained on all subjects except for one and then tested on that out-of-sample individual, and this was repeated for each individual), the accuracy reaches 83.9%. All results are summarized in Table 2.

**Table 2:** Classification accuracy for various neuroimaging markers (50% of the data are used as training set and the rest 50% as test set, except for the last column, where a leave-one-out cross-validation is used).

Neuroimaging markers	A: Community matrix *K*(400 edges)	B: Community Matrix *K*(50 edges)	C: Asymmetry *d*: 45-dimensional	D: Asymmetry*ρ*: 45-dimensional	ECross correlation matrix(400 edges)	B+D	A+D	A+D(Leaveoneout)
Predictionaccuracy(mean±std)	77.6% ±3.47%	73.2% ±3.89%	75.8%± 3.64%	75.5% ±3.62%	70.5%± 4.48%	76.9%± 3.59%	80.2% ±3.45%	83.9%
Sensitivity(mean)	77.1%	72.3%	75.2%	74.8%%	69.7%	75.4%	78.4%	82.5%
Specificity(mean)	78.0%	73.7%	76.3%	76.1%	71.1%	78.1%	81.6%	85%

The best results are achieved when we combine the features from the community matrix *K* and the asymmetry measure *ρ*. The accuracy of classification using SVM versus the number of edges selected from the community matrix *K* can be found in the Text S4.

## Discussion

### Brain Asymmetry in Epileptic Patients

Asymmetry is a fundamental feature of the human brain that has been shown to be altered in many neuropsychiatric disorders such as epilepsy [44], schizophrenia [45], and autism [46]. However, many work focus on brain asymmetry in terms of anatomical change. Currently, there is not much work on the asymmetry based on global functional connectivity, and little is known about how neuropsychiatric disorders like epilepsy can disrupt the asymmetry of bilaterally homologous brain regions at the level of functional brain network. From Fig. 4b we can see that the asymmetry of patients increases significantly in a large number of symmetric brain regions. In fact for all the 16 regions with significant difference across two groups (Fig. 4c only shows the most asymmetric 10 regions), the asymmetry of patients is found to be larger than that of the healthy controls. We find that the most different regions across the two groups revealed by asymmetry *ρ* and pair wise brain-region synchronization index *d* are quite consistent (see Figure 4b and Figure 5b), and are mainly distributed in temporal lobe and limbic system (8 regions), occipital lobe (5 regions), and parietal (1 region) and frontal lobe (1 region). This result suggests that regions in temporal and limbic lobes are more likely to be affected in epileptic patients. It is interesting that both measures rank the amygdala highly. The amygdala is primarily involved in emotional and social behavior like fear conditioning and face perception [47], and may be affected alone or in combination with other regions in temporal lobe epilepsy. We also note that among the most different 20 functional connectivities across the two groups, the right-hemisphere is more significant, accounting for 35% of the links (i.e., 7 links, see Table 1) in contrast to the 3 links that belong to the left-hemisphere. Our results suggest a potential correlation between the causes of epilepsy and the asymmetric global functional connectivity patterns in cerebral cortex, which is a highly sensitive neuroimaging marker and may shed light onto the neurologic nature of epilepsy. One reason for the observed functional asymmetry of the epileptic patients may be due to the large percentage of partial seizure patients. Since partial seizures are localized seizures that affect only one side of the brain, the level of functional asymmetry thus is expected to be higher. Whether this enhanced asymmetry at the network level is a common phenomenon for other neuropsychiatric disorders remains an interesting question.

Conceptually, the two asymmetry measures proposed in the paper capture different properties of the functional brain network. The global connectivity asymmetry *ρ* depicts the asymmetry of two bilaterally symmetric brain regions by their global connection profile; while pairwise brain-region synchronization *d* only characterizes the local synchronization between these two regions. Although the significant regions identified by these two measures are largely similar, the sensitivity of these two measures is different. We also find that the correlation between the global and local asymmetry measure varies with brain regions and across the two groups. It remains an interesting topic to see how these two measures are correlated.

### Influence of Medications

About 90% of the epilepsy patients in the present study are taking antiepileptic drugs. The drugs function by blocking sustained repetitive firing in individual neurons. Since the medications can affect both normal and abnormal regions, thus, it is possible that the distinct patterns of functional connectivity for patients may arise from the drugs. To clarify the origin of the observed difference across the two groups, we performed a cross-validation with the training set consisting of the patients using drugs plus the healthy controls (162 subjects), and the test set includes patients with no medications (18 subjects). We obtained 83.3% accuracy on the test group by SVM classifier using the same set of features as before, which indicates that there is no significant difference between patients with and without medication. We therefore conclude that the difference identified across two groups is mainly due to epilepsy itself rather than the antiepileptic drugs.

### High-dimensional Features and Pattern Classification

One characteristic of our classification approach is the high dimensionality of the neuroimaging markers, or features being adopted: the community matrix *K* (we select 400 edges in *K*), and the asymmetry *ρ* (45 dimensional), which characterize local and global properties of the whole network, respectively. The reason why such a high dimensional features are needed is due to the fact that the epileptic patients in our study are large in number and inevitably involve many different subtypes of epilepsy. Each different types of epilepsy are characterized by different traits. For example, epilepsies associated with distinct brain lobes may have different, altered, functional connections. Therefore the high classification accuracy in our case (>80%) is possible only when large number of functional connectivies (i.e., high-dimensional features) are used. In fact the predictive power is attributed primarily to the essential features that are not extremely high dimensional. For example, an averaged accuracy of 73.2% and 75.8% can be achieved, respectively, using 50 most discriminative edges from the community matrix *K* and 12 regions selected from asymmetry measure *d*, and the second feature seems more dominant in classification. The advantage of our global-feature approach is that it could potentially involve features for many subtypes of epilepsy and a further sub-classification is possible by simply using part of the features already found.

Here the functional connectivity was measured between each pair of brain regions comprising the AAL template that is rather coarse. It has been shown that the resolution of nodal parcellations can influence network properties [48], [49], which in turn may influence the classification. Theoretically, a higher resolution in parcellation may provide more spatially-accurate information regarding the altered functional connectivity. However, since the number of functional connectivity is about the square of the number of nodes, a high-resolution template thus can greatly increase the dimensionality of the problem that can hamper the performance of classifier. It is therefore reasonable to use a low-resolution parcellation first (such as the AAL template) to identify coarsely those brain regions whose functional connectivities have changed. Based on this a high-resolution parcellation may further be applied to spot the changes at a small scale. It remains our future work to use a high-resolution parcellation scheme in the classification task.

Many publications have reported the significantly changed functional networks at the group level (i.e., finding group difference), which is much simpler than a classification task at the individual level as we presented here. Usually, in order to achieve a high accuracy, highly sensitive neuroimaging markers are needed. For example, suppose there are 56 healthy controls and 50 patients having a link from temporal-pole-mid (left) to temporal-pole-mid (right), respectively, for the 180 subjects we studied. Hence the difference (56/80–50/100 = 20%) is significant. However, when it comes to classification using this feature, the accuracy is only (28+25)/90 = 59% (if 50% are selected as the test set), which is quite low. This simple example suggests that to achieve a high accuracy in classification is much harder than to find significantly changed links, and only those highly-sensitive features can contribute to a good performance. For highly heterogeneous data set (like the epileptic data used here which contains many subtypes), high-dimensional features are always necessary. Finally, the high accuracy in our classification here suggests that various types of epilepsy may share common characteristics: alteration in the pattern of functional integration among distributed brain regions, and an increase in brain asymmetry, which are well captured by the proposed community matrix K and asymmetry *ρ*. Finally it should be mentioned that the approach proposed in the paper is also of great relevance to electroencephalogram and magnetoencephalograph data analysis: our approach can be conveniently applied to such data. Previous studies in this field have used functional connectivity analysis as a diagnostic tool in patients suspected to have epilepsy [50].

Our current approach can be applied to a wider range of neuropsychiatric disorders such as Alzheimer’s disease, depression, schizophrenia, ADHD, etc., the diagnosis of which bears more clinical significance than epilepsy as the syndromes of these brain disorders are often not obvious especially at an early age. It remains an interesting question whether the neuroimaging markers proposed in this paper could be sensitive to other neuropsychiatric disorders.

## Supporting Information

Figure S1 **The plot of community matrix** ***K*** **under different** ***k*** **(** ***k*** **= 15, 20, 25, 30) for a healthy subject.**
(EPS)

Figure S2 **The Frobenius norms of the difference matrix between community matrix (** ***K_k_*** **) under various** ***k*** **and community matrix with** ***k =*** **30 (** ***K*** **_30_) versus the number of clusters** ***k*** **in** ***k*** **-means clustering.**
(EPS)

Figure S3 **The accuracy of classification using SVM versus the number of edges selected from the community matrix** ***K*** **(50% of the data are used as training set and the rest 50% as test set).**
(EPS)

Text S1 **Community matrix** ***K*** **under different** ***k.***
(DOCX)

Text S2 **Feature selection by sparse regression.**
(DOCX)

Text S3 **Standardized Euclidean Distance.**
(DOCX)

Text S4 **SVM classifier.**
(DOCX)
