# Supplementary material for: Pattern Classification of Large-Scale Functional Brain Networks: Identification of Informative Neuroimaging Markers for Epilepsy
Source: PLoS One. 2012 May 17;7(5):e36733. doi: 10.1371/journal.pone.0036733 (PMC3355144; doi:10.1371/journal.pone.0036733)
Supplement: Text S1 — Community matrix K under different k. (DOCX) [file pone.0036733.s004.docx]

**Supplementary Text S1**

**Community matrix *K* under different *k***

The community matrix *K* demonstrates quite consistent connectivity patterns (with only slightly different degrees of sparsity) for *k* within a wide range. From Fig. S1 we can see that the matrices *K* obtained at different *k* have very high correlation. The correlation coefficients among the four matrices shown in Fig S1 are: *ρ*(*K*_15_,*K*_20_)=0.90; *ρ*(*K*_15_,*K*_25_)=0.98; *ρ*(*K*_15_,*K*_30_)=0.95; *ρ*(*K*_20_,*K*_25_)=0.90;)=0.99; *ρ*(*K*_20_,*K*_30_)=0.90;)=0.96; *ρ*(*K*_25_,*K*_30_)=0.99, which indicate that community matrices *K* under different *k* contain similar information, i,e., connectivity patterns. The Frobenius norms of the difference matrix between *K_k_* and *K_30_* is also demonstrated in Fig. S2. From Fig. S1 and Fig. S2, which we can see that the community matrix *K* obtained under various *k* (ranging from 15 to 45) are very much alike.
