# Supplementary material for: Pattern Classification of Large-Scale Functional Brain Networks: Identification of Informative Neuroimaging Markers for Epilepsy
Source: PLoS One. 2012 May 17;7(5):e36733. doi: 10.1371/journal.pone.0036733 (PMC3355144; doi:10.1371/journal.pone.0036733)
Supplement: Text S2 — Feature selection by sparse regression. (DOCX) [file pone.0036733.s005.docx]

**Supplementary Text S2**

**Feature selection by sparse regression**

The community matrix *K* is a symmetric 90*90 matrix, with the entries in its upper (or lower) triangle constituting a 4005-dimensional feature vector *a*. By formulating the feature matrix of the training set (each row in the feature matrix is a 4005-dimensional vector *a* from one subject) and the label of subjects (1 for healthy and -1 for patients) into a linear regression, sparse regression returns a regression coefficient vector *x* (4005 dimensional), each entry of which (the absolute value) indicates the contribution of the corresponding feature to discriminating the two groups. It can provide effective feature selection even when the number of training subjects (90) is much lower than the number of features (4005).

The basic form of the regression is *y=Ax+ε* in which *A* is the data matrix, where each row is a 4005 dimensional vector *a* representing one subject, and each column represents a feature; *x* is regression coefficients; *y* is the label vector, with 1 representing healthy controls and -1 for epileptic patients. The basic idea of sparse linear regression, is to penalize the L_1_-norm *|x|_1_* of the regression coefficients *x* (i.e., min_x_ |Ax - y|^2^ + λ |x|_1_, where λ is the regularization parameter that controls the degree of sparsity, and a larger λ leads to *x* with more zeros, indicating that the corresponding features are not relevant). By solving the sparse regression problem, we obtain the regression coefficients *x* whose absolute value indicates the contribution of the corresponding edges to discriminating the healthy and epileptic groups.

It has been shown that sparse regression provides a consistent estimate even when the number of training subjects is much lower than the number of features. This is particularly valuable here as the number of subjects in training sets (90) is significantly lower than that of network features (4005). To further improve the stability of the results and enrich the feature selection, we apply a random sampling scheme by performing a series of regressions (200 times), each time on a randomly selected subset of features (20%), and then integrate the results. The advantage of sparse regression with random sampling in feature selection is that it can preserve a group of relevant features that, combined, will possess even higher discrimination power, rather than considering each feature separately (like independent multiple hypothesis test) .
