# Supplementary material for: Pattern Classification of Large-Scale Functional Brain Networks: Identification of Informative Neuroimaging Markers for Epilepsy
Source: PLoS One. 2012 May 17;7(5):e36733. doi: 10.1371/journal.pone.0036733 (PMC3355144; doi:10.1371/journal.pone.0036733)
Supplement: Text S3 — Standardized Euclidean Distance. (DOCX) [file pone.0036733.s006.docx]

**Supplementary Text S3**

**Standardized Euclidean Distance**

The standardized Euclidean distance between two time series *x* and *y* (row vector with j entries) can be written as:

$$d(x,y)=\sqrt{\sum_{j=1}^{J} (\frac{x_{j}}{s_{j}}-\frac{y_{j}}{s_{j}})^{2}}$$

where *s_j_* is the sample standard deviation of the *j*-th variable. For standardized Euclidean distance, each coordinate difference between *x* and *y* is scaled by dividing by the corresponding element of the standard deviation.
