# Supplementary material for: Pattern Classification of Large-Scale Functional Brain Networks: Identification of Informative Neuroimaging Markers for Epilepsy
Source: PLoS One. 2012 May 17;7(5):e36733. doi: 10.1371/journal.pone.0036733 (PMC3355144; doi:10.1371/journal.pone.0036733)
Supplement: Text S4 — SVM classifier. (DOCX) [file pone.0036733.s007.docx]

**Supplementary Text S4**

**SVM classifier**

We used Gaussian kernel in the SVM classifier. The kernel width *h* and the regularization parameter *C* in SVM are determined by standard 5-fold cross validation implemented in libsvm software package. More specifically, the candidate values for *C* is {0.001 0.01 0.1 1 10 100 1000}, that for the kernel width is *h_0_**{1/32 1/16 1/8 1/4 1/2 1 2 4 8 16 32}, where *h_0_* is the averaged pair wise distances between samples. In addition, the parameter λ in the regression is also determined by the cross validation.
